# An Autoscaling System Based on Predicting the Demand for Resources and Responding to Failure in Forecasting

**DOI:** 10.3390/s23239436

**Published:** 2023-11-27

**Authors:** Jieun Park, Junho Jeong

**Affiliations:** Department of Computer Science and Engineering, Dongguk University, Seoul 04620, Republic of Korea; 5656jieun@dgu.ac.kr

**Keywords:** cloud computing, edge computing, autoscaling, real-time processing, deep learning

## Abstract

In recent years, the convergence of edge computing and sensor technologies has become a pivotal frontier revolutionizing real-time data processing. In particular, the practice of data acquisition—which encompasses the collection of sensory information in the form of images and videos, followed by their transmission to a remote cloud infrastructure for subsequent analysis—has witnessed a notable surge in adoption. However, to ensure seamless real-time processing irrespective of the data volume being conveyed or the frequency of incoming requests, it is vital to proactively locate resources within the cloud infrastructure specifically tailored to data-processing tasks. Many studies have focused on the proactive prediction of resource demands through the use of deep learning algorithms, generating considerable interest in real-time data processing. Nonetheless, an inherent risk arises when relying solely on predictive resource allocation, as it can heighten the susceptibility to system failure. In this study, a framework that includes algorithms that periodically monitor resource requirements and dynamically adjust resource provisioning to match the actual demand is proposed. Under experimental conditions with the Bitbrains dataset, setting the network throughput to 300 kB/s and with a threshold of 80%, the proposed system provides a 99% performance improvement in terms of the autoscaling algorithm and requires only 0.43 ms of additional computational overhead compared to relying on a simple prediction model alone.

## 1. Introduction

In recent years, the convergence of edge computing and sensor technologies has emerged as a major frontier for the augmentation of real-time data processing. This includes applications in which the effective processing of data collected from a multitude of sensors is of the utmost importance, encompassing various fields, such as transportation, healthcare, and emergency response, all of which are based on 6G communication [1]. One example is the use of intelligent traffic control systems, wherein high-speed autonomous vehicles on highways require real-time interaction with their surroundings, other vehicles, and humans. Unfortunately, a considerable portion of the existing artificial intelligence (AI)- and cloud-based research centers for static data processing have failed to address the requirements of real-time processing. Recent studies have shown that AI solutions implemented in edge networks—wherein intelligent prediction, reasoning, and decision-making tasks are performed—can decrease service response times and provide genuine real-time services [2,3,4].

As the demand for these applications increases, problems related to resource allocation and potential resource overload have become evident [5,6]. The basic premise of this technology is to facilitate rapid response times by localizing data processing near the Internet of Things (IoT) devices. However, the increased demand and potential resource overload can lead to serious problems, such as increased response latency and system failure.

One way to solve this problem is through container orchestration. Kubernetes is the most popular container orchestration platform and offers the horizontal pod autoscaling method, designed to dynamically fine-tune resource allocation based on current utilization metrics [7]. Nonetheless, this post-processing method involves time-consuming state validation and the creation or removal of resources when predefined thresholds are exceeded, which can introduce system-overload-related problems. Concurrently, research efforts have explored the use of deep learning techniques for the proactive prediction of resource allocation [5,8,9,10,11,12]. Most of these studies engage in time-series forecasting by leveraging log data such as HTTP requests and CPU utilization. A major benefit of this predictive approach is that resource tuning can be executed proactively, thereby mitigating the risk of overload. However, it is crucial to acknowledge that resource overload problems can still exist if the prediction model is unstable.

This paper presents a system architecture for the operationalization of this methodology within a real container orchestration system. To address these problems for effective resource provisioning, we propose a novel resource demand prediction method that combines deep-learning-based forecasting with subsequent monitoring and dynamic resource adjustment to overcome forecasting failures. This predictive capability aims to preemptively adjust resources in response to evolving demands, thereby ensuring efficient resource utilization within the dynamic landscape of IoT-enabled edge computing. In other words, the proposed system aims to pre-emptively forecast the resource demand to facilitate proactive resource allocation before overload scenarios manifest. The proposed system provides a basic predictive capability by forecasting the future resource demand based on historical time-series data using deep learning algorithms combined with periodic inspection phases, to reduce the uncertainty inherent in predictive modeling. These inspection phases enable dynamic adjustments to be made if the initially allocated resources are insufficient to meet the actual demand.

The contributions of this research can be summarized as follows:

(1) The development of a resource forecasting method that enables proactive resource adjustments before overload situations occur;
(2) An intermediate inspection process based on network throughput for adjustment when the actual demand differs from deep-learning-based forecasts;
(3) A 99% improvement in the elastic speedup value through actual system implementation by applying the proposed methodology in an authentic container orchestration environment, under experimental conditions with the Bitbrains dataset, setting the network throughput to 300 kB/s and using a threshold of 80%.

The remainder of this paper is organized as follows. Section 2 reviews related research, both in terms of resource prediction and post-processing methods. In Section 3, we present our proposed methodology and elaborate on each step of the method. Section 4 provides insights into the experiments conducted to evaluate the proposed method, along with an analysis of the experimental results. Finally, Section 5 concludes the study and summarizes the key findings and implications.

## 2. Related Work

In this section, we present an overview of studies relevant to the research presented here. These studies can be classified into two primary categories—demand forecasting and post-processing. Within the purview of demand forecasting, our research delves into the distinctions between univariate predictions (which focus on the consideration of a single variable) and multivariate predictions (which entail the use of multiple variables), all of which are grounded in the analysis of time-series data. Moreover, a bidirectional long short-term memory (Bi-LSTM) [13] model is introduced, which serves as the designated prediction model employed in this study.

### 2.1. Demand Forecasting Using Deep Learning

Time-series data constitute a sequential collection of records ordered by time and serve as a pivotal resource for the analysis of underlying trends, patterns, and recurring phenomena. Their primary value is the capacity to anticipate future trends and discern changes over time. Within the context of resource demand forecasting, this analytical approach entails a systematic examination of periodic patterns grounded in data sources, such as the temporal evolution of HTTP requests, network traffic, and CPU utilization, to provide insights into near-term demand fluctuations. This predictive approach can be segregated into two distinct categories—univariate predictions (which rely on a solitary data variable) and multivariate predictions (which harness multiple data variables in tandem).

Prachitmutita et al. [8] introduced an innovative autoscaling framework underpinned by long short-term memory (LSTM) [14] and multilayer perceptron (MLP) models, primarily designed for univariate datasets, with a specific focus on the FIFA World Cup dataset [15], a valuable repository of HTTP request data. The results revealed that the LSTM model outperformed the MLP model, with greater precision in terms of workload prediction.

Mahmoud et al. [9] harnessed HTTP request data from the FIFA World Cup dataset [15] for predictive purposes. Their study compared the LSTM model with the autoregressive integrated moving average (ARIMA) model, culminating in the observation that the ARIMA model exhibited superior accuracy, although the LSTM model demonstrated a remarkable advantage in terms of prediction speed, being approximately 100 times faster.

Zhu et al. [10] investigated workload prediction by leveraging the CPU workload dataset from Alibaba cluster-trace-v2018 [16] and Dinda [17]. They proposed an inventive method using an LSTM-based encoder–decoder network augmented by an output layer. A comparative evaluation demonstrated that their method outperformed both the ARIMA and LSTM models, as evidenced by the improved root mean squared error (RMSE) scores.

Yoo et al. [11] conducted a predictive study employing both the NASA dataset [18] and the FIFA World Cup dataset [15], employing a Bi-LSTM [13] model tailored to time-series forecasting. Their model not only exhibited expedited predictions but also demonstrated enhanced accuracy when juxtaposed against the conventional LSTM and ARIMA models.

Xu et al. [12] introduced an efficient supervised-learning-based deep neural network (esDNN) method designed for cloud workload prediction in the domain of multivariate datasets. Their research drew from a realistic trace dataset derived from the Alibaba [16] and Google Cloud data centers [19], and the esDNN model yielded a noteworthy reduction in the mean square error (approximately 15% lower than when employing the GRU alone).

Yoo et al. [11] introduced a novel methodology using the GWA-T-12 dataset [20], in which they judiciously selected memory usage and network reception data with the highest correlation as pertinent features, and subsequently trained a bi-LSTM model. Their method, characterized by the incorporation of multivariate data, demonstrated remarkable performance superiority over univariate data analysis methods. Furthermore, when compared against other deep learning algorithms—such as LSTM and CNN-LSTM [21] algorithms—the proposed model consistently exhibited better performance.

Building on these foundational studies, this study advances the field by developing a prediction algorithm.

### 2.2. Post-Processing Algorithm

A noteworthy post-processing algorithm is the horizontal pod autoscaling algorithm proposed by Kubernetes [7]. This algorithm determines the optimal allocation of resources by assessing the ratio between the desired and current metric values, thereby enabling the dynamic adaptation of resource allocation in response to demand fluctuations. Nonetheless, this algorithm has limitations, primarily because it relies on threshold exceedances to trigger adjustments, which can lead to delays until the adjustments are fully implemented. Moreover, in edge computing environments characterized by heterogeneous resource demands across nodes, the algorithm falls short because it lacks the ability to intelligently allocate resources considering these variations.

To overcome these limitations, an alternative method has emerged that entails the allocation of additional pods to worker nodes experiencing a higher demand, as opposed to indiscriminately assigning pods to any available node upon a surge in cluster traffic. Although this approach retains its post-processing nature, it brings about substantial enhancements in terms of response time and throughput by allocating additional resources to nodes grappling with elevated request volumes. Moreover, it offers the advantage of cost efficiency [22].

**Figure 1:**
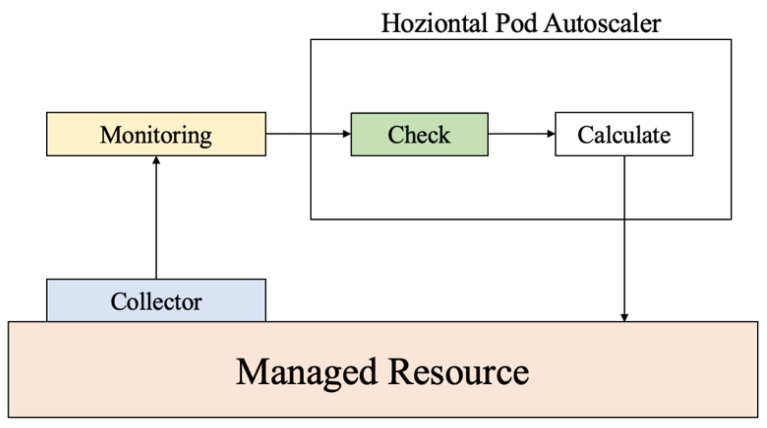
Structure of the horizontal pod autoscaling system in Kubernetes.

The architectural framework, as shown in Figure 1, serves as the foundational infrastructure underpinning the system developed within the scope of this study, leveraging Kubernetes’ horizontal pod autoscaler system. Metrics encompassing network traffic and CPU utilization gleaned from the cluster are diligently gathered by the collector, after which these metrics are relayed to the system, positioned externally to the cluster, and facilitated by a monitoring server. The core system function involves scrutinizing whether the received metrics surpass predefined thresholds. In the event of threshold exceedance, the system computes the required number of nodes, based on which it orchestrates resource adjustments within a cluster.

### 2.3. Bi-LSTM Model for Demand Prediction

**Figure 2:**
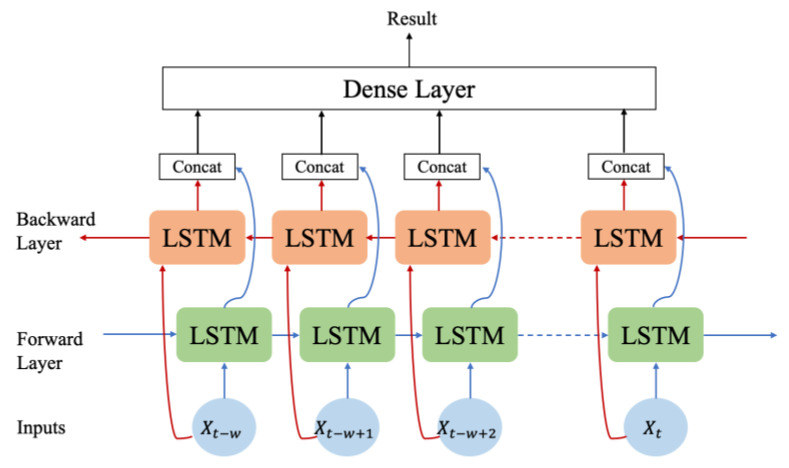
Structure of the Bi-LSTM model.

In the context of our time-series forecasting task, the proposed system employs a Bi-LSTM model, as shown in Figure 2, which is an extension of the LSTM architecture. The Bi-LSTM model represents a specific recurrent neural network (RNN) category adept at processing sequential data by bidirectionally propagating information, allowing for the incorporation of past context into the current state.

The LSTM was initially introduced to address the issue of long-term dependency, which can often plague standard RNNs. The Bi-LSTM model builds upon the LSTM framework and enhances its capabilities by enabling a bidirectional information flow. The Bi-LSTM model comprises four critical components—the input gate (1), forget gate (2), output gate (4), and cell state (3). Here, a concise description of the respective functions follows.

(1) Input Gate: The input gate determines the pertinent information to be retained based on the current input and the prior state. This decision is made using a sigmoid function. The output value of the input gate is a crucial determinant in shaping the cell state.
(2) Forget Gate: The forget gate plays a pivotal role in deciding which information to discard from the previous state. It considers both the prior state and current input, and its computations are governed by the sigmoid function.
(3) Cell State Update: The cell state update process involves the transition of the cell state of the previous time step to the current time step. Subsequently, a new cell state is calculated by integrating the inputs from the input gate and the current input. This operation plays a key role in storing and updating the network memory.
(4) Output Gate: The responsibility of the output gate is to determine the information that should be extracted from its current state. This plays a key role in shaping the hidden state in (5) for the current timestep.
(5) Hidden State: The hidden state is computed by combining the gate output and current cell state. This composite state encapsulates the network representation of the current time step and serves as the primary output of the model.

In summary, the Bi-LSTM model built on LSTM fundamentals excels at capturing complex dependencies in sequential data by processing information bidirectionally through these meticulously orchestrated components, ultimately making it an excellent choice for time-series forecasting tasks.

(1) $$i_{t}=\sigma_{g}(W_{i}x_{t}+U_{i}h_{t-1}+b_{i})$$

(2) $$f_{t}=\sigma_{g}(W_{f}x_{t}+U_{f}h_{t-1}+b_{f})$$

(3) $$c_{t}=f_{t}\cdot c_{t-1}+i_{t}\cdot \sigma_{c}(W_{c}x_{t}+U_{c}h_{t-1}+b_{c})$$

(4) $$o_{t}=\sigma_{g}(W_{o}x_{t}+U_{o}h_{t-1}+b_{o})$$

(5) $$h_{t}=o_{t}\cdot \sigma_{h}(c_{t})$$

Table 1 summarizes a comparison of recent studies related to the proposed approach. Related studies using multiple performance metrics—such as CPU utilization, memory usage, disk I/O, and network throughput—are limited [21]. The data type indicates the classification of the time-series data used, which can be categorized into two types—univariate and multivariate data. *ML/DL* signifies whether the method is based on machine learning, deep learning, or another methodology, whereas *Method* denotes the technique employed. *Resource Allocation Approach* denotes whether the implemented approach aims to make forecasts or merely perform checks.

In most prior studies, resource adjustments were predominantly performed through preemptive resource allocation based on predictions or by surpassing a threshold based on current usage. Predictive methods can be categorized into machine-learning-based methods—such as ARIMA and MLP—and deep-learning-based methods—which use LSTM and neural networks. Although deep-learning-based models tend to outperform machine-learning-based models, both methods share the limitation that they require re-adjustments if the predictions fail [5,10,11].

Compared to a forecast-driven prediction strategy, post-processing using the intermediate check method offers the benefit of computing the necessary resources once a certain threshold is surpassed and making precise adjustments. Nonetheless, it has the drawback of requiring a cooldown period for the creation of new VMs, during which processing must rely on existing resources. Consequently, in this study, we devised a system that addressed the limitations of both methods by integrating an intermediate check process that used a predictive model as a foundation and a post-processing method between forecasts.

## 3. Methods

### 3.1. System Architecture

**Figure 3:**
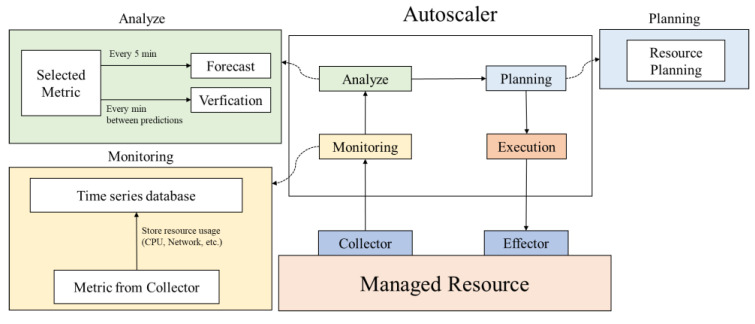
Structure of the overall autoscaling system.

Figure 3 shows the system architecture and demonstrates the application of the proposed model in a practical container orchestration system. Metrics such as network reception and memory usage are collected from a managed cluster through collectors and sent to a time-series database, such as Prometheus [23]. The collected data are then transmitted to an autoscaler located outside the cluster. At this stage, the proposed system predicts and plans resource demand adjustments. Following this phase, adjustments are made based on the established plan. When configuring the prediction and inspection cycles, it is essential to consider factors such as the workload of the managed cluster and the time required for implementation.

If the cycle duration is set too short, frequent incorrect adjustments can occur owing to rapid fluctuations. Moreover, there is a risk of making readjustments before observing the effects of previous adjustments [6]. To address these concerns, there is a waiting period after executing the adjustments that allows for a brief pause before initiating inspections. After resource adjustments are made during the planning phase, there is a one-minute interval during which no further action is taken. The prediction cycle occurs every five minutes, with the timing based on the reference point following the waiting period after the initial prediction. Over the subsequent five minutes leading up to the next prediction, the proposed system engages in an interim inspection, and resource adjustments are performed if deemed necessary. This interim inspection process occurs every minute. Even if actual adjustments are enacted during this phase, another waiting period is incorporated, allowing time for the effects of these adjustments to materialize.

**Figure 4:**
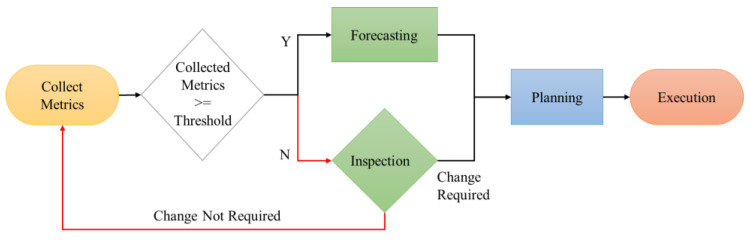
Flow chart for the entire system.

Figure 4 shows a flowchart illustrating the system’s operation. Initially, information on server usage is collected from the metric server. If at least 5 min elapse, predictions are made; otherwise, inspections are performed. Subsequently, the required number of VMs is calculated, and a plan for system adjustments is formulated. Finally, the system performs modifications according to the plan and concludes its operation.

### 3.2. Forecasting Stage

During the forecasting stage, the proposed system performs periodic workload predictions. In this study, the Bi-LSTM model was selected for our prediction model, as described in Section 2.3. The model leverages both antecedent and subsequent data at the present time step. This unique approach permits the use of future information, thereby contributing to enhanced prediction accuracy.

Upon the collection and incorporation of metrics into the system, the system harnesses our pretrained model to formulate predictions. These predictions serve as the foundation for subsequent actions. Specifically, the planning algorithm (elaborated upon in Section 3.4) computes the requisite quantity of resources essential for accommodating the anticipated workload guided by the predicted outcomes. This seamless integration of forecasting and resource planning is a pivotal strategy that enables the judicious allocation of resources and the optimization of system performance.

### 3.3. Proposed Interim Inspection Stage

Relying solely on a prediction algorithm would be ideal in the pursuit of flawless predictions. However, these aspirations are often unattainable. Hence, an interim inspection algorithm was introduced, as presented in Algorithm 1, assuming the role of assessing whether the existing quantity of resources was sufficient to manage the current network reception input. This algorithm draws inspiration from Kubernetes’ horizontal pod autoscaling algorithm and hinges on the assessment of the ratio between the desired and prevailing metric values. Moreover, the proposed algorithm integrates considerations for the maximum network throughput of a VM and threshold values to ensure adaptability to fluctuations in the network throughput attributed to VM performance variations, thereby facilitating the calculation of an appropriate network throughput value consistent with the defined criteria.

In the proposed interim inspection phase, four essential inputs are required—the input denoting the presently received network throughput; ${now}_{vm}$ denoting the number of currently active VMs; threshold denoting the VM utilization limit; and ${network}_{max}$ denoting the maximum network throughput achievable on a VM. Additionally, the system computes network, which denotes the network throughput constraints. This value is derived by multiplying the maximum network throughput by the threshold value. Subsequently, the requisite number of VMs can be ascertained through the following formula: ${required}_{vm}=ceil[{now}_{vm}\times (input/network)]$. If the computed number of required resources deviates from the current resource count (i.e., ${now}_{vm}$), it serves as an indicator of the potential scaling requirements, either upscaling or downscaling. In such cases, the algorithm triggers the planning function and initiates a new planning phase in alignment with the observed resource requirements.
**Algorithm 1** Interim Inspection   **Input:** input//Currently received network throughput   ${now}_{vm}$//Number of VMs currently in use   threshold//Limit of VM utilization   ${min}_{vm}$//Number of minimum VMs   ${network}_{max}$//Maximum network throughput on a VM   **Output:** Scheduled planning actions 1: Initialization 2: $network={network}_{max}\;*\;threshold$ 3: ${required}_{vm}=ceil[{now\;}_{vm}*\;(input/network)]$ 4: $if\;{required\;}_{vm}\;is\;not\;{now}_{vm}then$ 5:    $Planning(input,\;{now}_{vm})$ 6: else 7:     Sleep 8: end if 

Before delving to the planning phase, it is important to clarify the underlying rationale behind the adoption of periodic predictions and interim inspection procedures. As listed in Table 2, the time required to conduct periodic predictions and interim inspections can be quantified. Considering the time spent on these two activities while excluding the fixed time allotted for resource adjustments, there is a substantial contrast. Periodic predictions, conducted at 5-min intervals, require approximately 19.46 ms, whereas interim inspections require a mere 0.4528 ms (a 43× difference). This stark disparity underscores the efficiency achieved by incorporating interim inspections in tandem with periodic predictions. Nonetheless, it is important to acknowledge that both time intervals fall within the one-second range, rendering any noticeable difference in practical terms negligible. Moreover, the impetus behind instituting interim inspection lies in the intrinsic divergence between the prediction outcomes and actual resource demand. As discussed in the forthcoming Section 4.4, the prediction results often exhibit disparities, particularly during periods characterized by heightened demand. Consequently, even if predictions are made at frequent intervals, their consistent inadequacy, particularly during high-demand scenarios, can lead to an amplified overhead associated with resource adjustments. Consequently, the expected benefits of resource preparation remain unknown.

However, a paradigm shift can be accomplished by subjecting the actual resource demand to interim inspection and subsequently executing resource adjustments solely when warranted. This provides an avenue for the optimization of resource management by mitigating waste and promoting efficient resource utilization. While an ideal scenario would entail the accurate provisioning of all resources grounded solely in perfect predictions, reality underscores the fallibility of predictions. In this context, the incorporation of an interim inspection process not only enhances the prediction accuracy but also augments the resource efficiency. This, in turn, provides more fitting predictions for the actual resource demand, thereby fortifying the effectiveness of resource management.

### 3.4. Planning Stage

Subsequent to the execution of forecasting or post-processing operations, periodic verification of the actual resource demand is of great importance. In this context, Algorithm 2 serves as a manifestation of the planning phase and is dedicated to ascertaining the requisite number of resources, which is a prerequisite for both prediction and periodic verification scenarios.

Within the planning stage, a set of four vital inputs is mandated—the input denoting the anticipated network workload derived from the prediction; now_vm denoting the prevailing count of active VMs; threshold denoting the threshold value governing VM utilization; and ${network}_{max}$ denoting the upper limit of the network throughput achievable on a VM.
**Algorithm 2** Planning   **Input:** input//predicted network workload   ${now}_{vm}$//Number of VMs currently in use   threshold//Limit of VM utilization   ${min}_{vm}$//Number of minimum VMs   ${network}_{max}$//Maximum network throughput on a VM   **Output:** Scheduled scaling actions 1: Initialization 2: $network={network}_{max}\;*\;threshold$ 3: ${next}_{vm}=ceil[{min\;}_{vm}*\;(input/network)]$ 4: $if\;{next\;}_{vm}>{now}_{vm}\;then$ 5:   $SCALE\_UP({next}_{vm})$ 6: $else\;if\;{next}_{vm}<{now}_{vm}\;then$ 7:   $SCALE\_DOWN({next}_{vm})$ 8: else 9:   Sleep 10: end if 

In Line 1, the proposed system computes network, denoting the network throughput constraint, computed as the product of the maximum network throughput and the threshold value. In Line 2, the essential number of VMs is determined using the following formula:$\;{next}_{vm}=ceil[{now}_{vm}\times (input/network)]$. In Lines 3–6, the calculated number of required resources diverges from the current resource count (i.e., ${now}_{vm}$), and the system promptly initiates either upscaling or downscaling, depending on the prevailing state of the system.

In Line 7, if the calculated figure aligns with the existing resource count, the system maintains its current resource allocation configuration. This planning algorithm exemplifies the adaptability of the system, ensuring the dynamic adjustment of resource allocation to harmonize with forecasted or actual workloads. This adaptive approach culminates in the optimization of resource utilization and augments the overall performance of the system.

## 4. Results

### 4.1. Dataset

The GWA-T-12 Bitbrains dataset [20], generously provided by Bitbrains, served as the primary data source for both training and testing in this study. This extensive dataset encompasses performance metrics gathered from 1750 VMs situated within the distributed data centers of Bitbrains. The dataset is bifurcated into two distinct files—namely, the “FastStorage” and “Rnd” files. The “FastStorage” file comprises data originating from VMs tasked with hosting frequently accessed programs, while the “Rnd” file encapsulates data emanating from less frequently accessed and lower-performance VMs. Notably, for the scope of this study, the “FastStorage” dataset was employed exclusively.

The dataset comprised a diverse array of performance metrics encompassing parameters such as CPU usage, CPU utilization, memory usage, disk read throughput, disk write throughput, network reception throughput, and network transmission throughput. In the context of model training, the memory usage and network reception throughput metrics were selected. This selection was underpinned by the highest Pearson correlation coefficient, denoting a robust and substantial relationship with the target variable [5]. Prior to commencing model training, the dataset was normalized, standardizing its values to fall within the [0, 1] range, thereby ensuring uniformity in the data distribution. Subsequently, the dataset was partitioned, allocating 80% to train the model while reserving the remaining 20% for the critical task of evaluating the model’s performance. This partitioning strategy enabled the assessment of the model’s capacity to effectively generalize unknown data instances.

### 4.2. Experiment Details

First, a training phase was initiated to develop a predictive model. For training purposes, we selected the network received throughput and memory usage as they exhibited the highest correlation value, and the training process employed a Bi-LSTM model, following the tuned hyper-parameter specific configuration detailed in Table 3 [5]. Initially, the training was scheduled to encompass 100 epochs; however, to optimize the efficiency, we introduced an early stopping mechanism. When triggered, this mechanism ceased the training process if it discerned no significant improvement, thereby conserving the computational resources. This strategy is particularly advantageous for low-performance VMs.

Second, we conducted a comparative analysis of the outcomes obtained by implementing the proposed algorithm on the trained Bi-LSTM model and those derived without its application. To facilitate this assessment, we leveraged system-oriented elasticity metrics tailored to gauge the efficiency of resource provisioning. During the prediction phase, coupled with an interim inspection, a cooldown period of 1 min was imposed after any resource adjustment. This precautionary measure aimed to forestall additional modifications before the effects of the preceding actions became discernible. Additionally, predictions occurred at 5-min intervals, with interim inspection routines executed every 1 min.

Conversely, in the prediction phase without interim inspection, predictions transpired every minute if no resource adjustments occurred. In the non-autoscaling scenario, the number of autoscalers was fixed at 2, with a specified network throughput threshold per VM set at 0.8. The maximum network throughput was 300 kB/s. Notably, the network number was intentionally kept minimal, accentuating the magnitude of fluctuations in the required number of VMs, thus facilitating the performance assessment.

### 4.3. Evaluation Metrics

To gauge the efficacy of resource provisioning, we employed the efficiency criteria prescribed by the Standard Performance Evaluation Corporation (SPEC) research organization [24], with the computation of efficiency metrics conducted in accordance with Equations (6)–(10).

(6) $$\theta_{u}=\frac{100}{T}\Sigma_{t=1}^{T}\frac{max\left(d_{n}-p_{n},0\right)}{d_{n}}\Delta t$$

(7) $$\theta_{o}=\frac{100}{T}\Sigma_{t=1}^{T}\frac{max\left(p_{n}-d_{n},0\right)}{d_{n}}\Delta t$$

(8) $$\tau_{u}=\frac{100}{T}\Sigma_{t=1}^{T}max(sgn\left(d_{n}-p_{n}\right),0)\Delta t$$

(9) $$\tau_{o}=\frac{100}{T}\Sigma_{t=1}^{T}max(sgn\left(p_{n}-d_{n}\right),0)\Delta t$$

(10) $$\epsilon_{n}={\left(\frac{\theta_{U,n}}{\theta_{U,a}}\cdot \frac{\theta_{O,n}}{\theta_{O,a}}\cdot \frac{\tau_{U,n}}{\tau_{U,a}}\cdot \frac{\tau_{O,n}}{\tau_{O,a}}\right)}^{\frac{1}{4}}$$

The under-provisioning resource metric ($\Theta_{u}$) serves as an indicator of the extent to which the allocated resources fall short of fulfilling the genuine demands of the system. Essentially, it quantifies the frequency at which a system encounters resource deficiencies, leading to performance degradation. A higher $\Theta_{u}$ value signifies a higher incidence of under-provisioning, potentially resulting in bottlenecks or reduced user satisfaction. Conversely, the over-provisioning resource metric ($\Theta_{o}$) assesses the degree to which resources are allocated in excess of the system’s actual requirements. This metric sheds light on scenarios in which resources are wasted because of overallocation. A higher $\Theta_{o}\;$value indicates a prevalence of over-provisioning, leading to suboptimal resource utilization.

The metrics denoted as “duration the system is under-provisioned” ($\tau_{u}$) quantify the cumulative period during which the system operates in an under-provisioned state. This measurement encapsulates the total time required by the system to cope with insufficient resource allocation, potentially resulting in performance bottlenecks and user dissatisfaction. By contrast, the metrics labeled “duration the system is over-provisioned” ($\tau_{o}$) calculate the cumulative period during which the system contends with an excess of resources. This metric highlights instances of resource inefficiency and suboptimal resource utilization.

Elastic speedup ($\epsilon_{n}$) denotes a metric devised to assess the performance enhancement of a specific approach relative to a scenario without autoscaling. This metric is particularly valuable in comparing two distinct strategies—that is, one incorporating autoscaling (denoted as a) and another devoid of autoscaling (referred to as n). The objective is to quantify the degree to which autoscaling improves the performance in comparison with a non-autoscaling scenario. By evaluating the system-oriented metrics such as $\Theta_{u}$, $\Theta_{o}$, $\tau_{u}$, and $\tau_{o}$ of method “a”, we can assess its efficiency relative to the baseline “n” scenario. A fundamental step involves computing the geometric mean of the ratios between the paired metrics. A value exceeding one indicates that the proposed method surpasses the non-autoscaling scenario, signifying a positive performance gain. Conversely, a value below 1 suggests that the proposed method underperforms compared to the non-autoscaling scenario.

### 4.4. Experimental Results

#### 4.4.1. Prediction Performance Results

**Figure 5:**
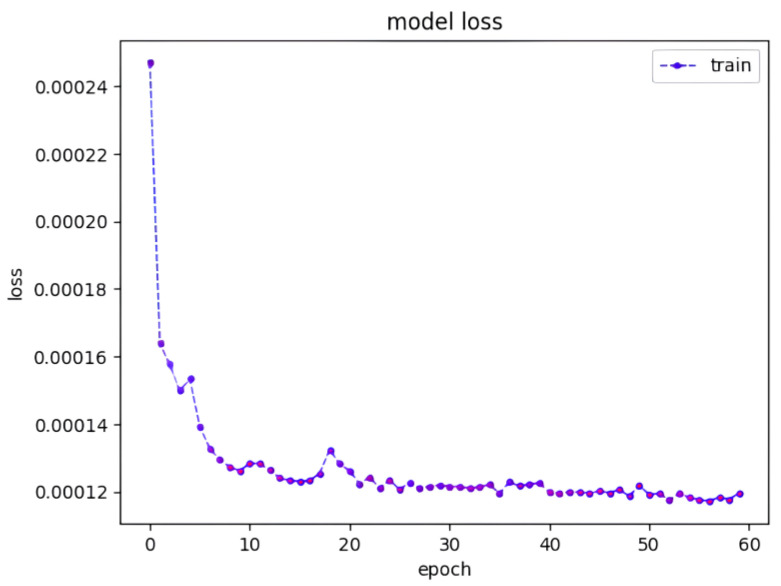
Model loss for the training phase.

**Figure 6:**
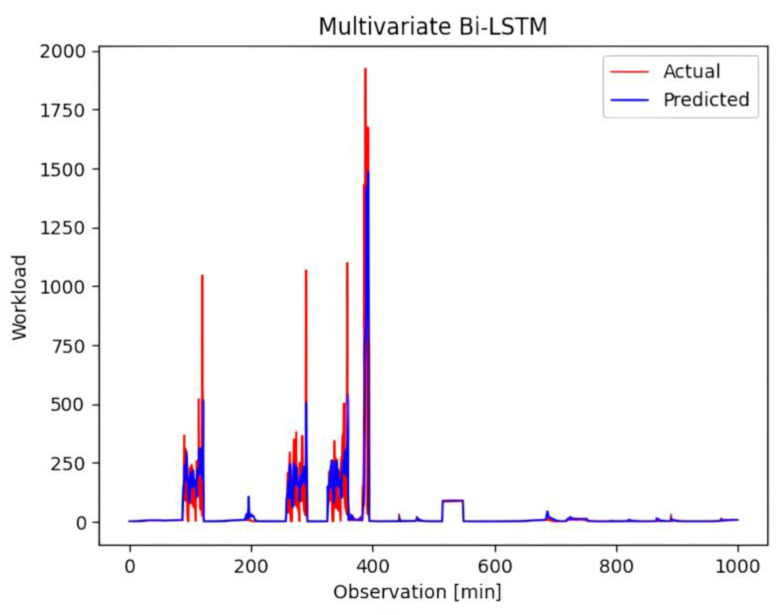
Predicted workload tested against the trained model.

The training progress is visually represented in Figure 5, which shows a consistent reduction in loss as the training epochs advance. To provide additional insight into the performance of the model, Figure 6 shows the predicted workload when subjected to testing using the trained model. Although the predicted workload is consistent with the underlying trend, instances of prediction failure can be highlighted, particularly when the incoming workload reaches elevated levels.

#### 4.4.2. Autoscaling Results and Comparison

**Figure 7:**
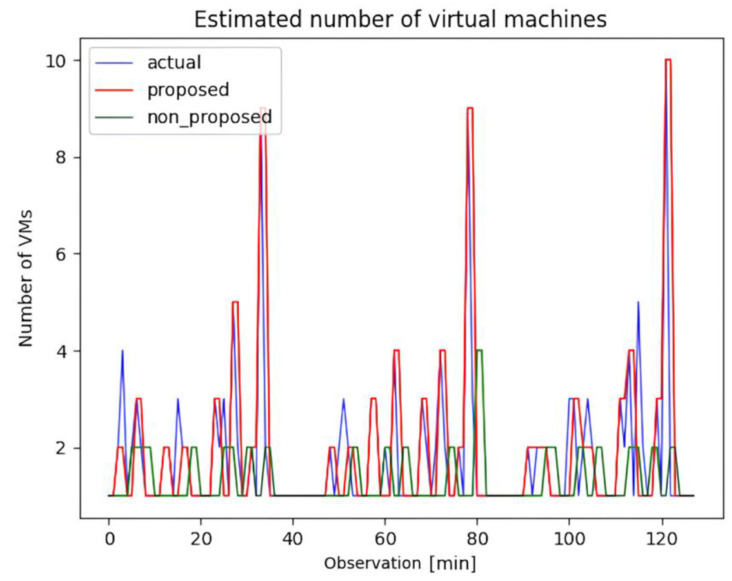
Estimated number of virtual machines with autoscaling: comparison between ground truth, autoscaling with proposed post-processing algorithm, and autoscaling by forecasting alone algorithm.

Figure 7 demonstrates the results of the proposed approach that combines forecasting and inspection, in comparison to the approach that solely employs simplistic forecasting. The result expresses the number of VMs with three colored lines, blue, red, and green, and the blue line represents the actual number of VMs. Specifically, the red line illustrates the number of VMs acquired through the application of the autoscaling model coupled with our interim inspection algorithm. By contrast, the green line shows the VM count achieved exclusively through the application of the autoscaling model, excluding the inspection algorithm. These visualizations provide insights into the impact of the interim inspection algorithm on the performance of the autoscaling model.

Additionally, Table 4 provides a comprehensive comparative analysis of three distinct methodologies—that is, no autoscaling, autoscaling without interim inspection, and autoscaling with the interim inspection algorithm. A thorough examination of Table 4 reveals that autoscaling using the proposed algorithm outperforms the alternative methods. This superiority is underscored by the smaller over-provisioning metric values compared with the other methods. Furthermore, it boasts superior elastic speedup ($\epsilon_{n}$) values, with a notable increase of 99% (2.31 compared to 1.16). This outcome accentuates the fact that the autoscaling method using the proposed algorithm achieves a more substantial autoscaling gain than the version lacking the algorithm. The observed disparities in the under-provisioning values within the context of autoscaling without the proposed algorithm can be ascribed to the inclination toward low-workload predictions. Moreover, when estimating the number of VMs to accurately represent the ever-changing real-time demand, the cooling-down period is not considered. Consequently, this scenario exhibits higher volatility in its values than the other scenarios, leading to inferior outcomes.

## 5. Discussion

The use of predictive models for autoscaling in real-time latency-sensitive IoT environments is important. This study leveraged a deep learning model to predict resource demands with the added implementation of intermediate verification steps in the event of prediction failures. This approach aims to mitigate the limitations of relying solely on predictive systems.

However, there are several problems associated with the methods described. First, the dataset used in this study did not represent applications that were heavily reliant on GPU and CPU resources. The data used in this study primarily pertained to the correlation between network throughput and memory usage, which were the most significant factors. If the dataset had encompassed applications that predominantly relied on GPU or CPU resources, adjustments to the algorithm and the choice of appropriate metrics would have been necessary. However, this issue is not a factor that significantly changes the proposed framework; it simply means that there may be discussions on evaluation methods for key metrics at each stage. Therefore, one can expect future improvements to methods that are effective even for datasets that rely on GPU and CPU resources.

Second, the autoscaling system in this study only considered scenarios in which resources were 100% available. It did not address the cases in which certain VMs had limited availability. Consequently, methods of calculating the overall system capacity by assessing the availability of VMs are required. Thus, the current system can adequately adjust resource allocation (even as resource availability increases), but further enhancements are essential to improve the efficiency and stability.

## 6. Conclusions

In this study, we introduced and implemented an interim inspection algorithm to mitigate the disparities between the predicted and actual demand. Following the prediction phase, this algorithm periodically assessed the demand, and, if disparities emerged, post-processing solutions were initiated. Notably, the proposed algorithm operated with a time requirement that was approximately 43 times shorter than the prediction process itself. By extending the prediction cycle and integrating the algorithm within an extended timeframe, the overhead imposed on the system was greatly reduced. Furthermore, this study recognized that exclusive reliance on prediction models for demand estimation could result in errors. To address this concern, an interim inspection was conducted to identify and rectify these errors. Notably, when prediction was combined with interim inspection, there was a substantial 99% improvement compared to the method without the proposed algorithm. When equipped with the proposed algorithm, the efficiency of the system surpassed that of systems that lacked optimization measures. Using this methodology, we aim to achieve the efficient provisioning of server resources for tasks that demand seamless real-time operations.

It is important to emphasize that this study primarily focused on assessing the overall demand for the entire cluster and performed post-processing accordingly. However, this study did not explicitly account for variations in demand among individual nodes within a cluster. Consequently, it did not address scenarios in which specific high-demand nodes might encounter resource allocation challenges. Future research should explore strategies that incorporate per-node demand considerations, thereby enabling more precise prediction and post-processing techniques to address demand variations at the node level.

## Figures and Tables

**Table 1:** Comparison of autoscaling- and resource-estimation-related studies in cloud computing.

Ref.	Performance Metrics	Data Type	ML/DL	Method	Resource Allocation Approach
2018, [8]	HTTP request	Univariate	Both	LSTM and MLP	Forecasting
2019, [9]	HTTP request	Univariate	DL	LSTM	Forecasting
2019, [10]	CPU utilization	Univariate	DL	LSTM-based encoder–decoder	Forecasting
2022, [11]	HTTP request	Univariate	DL	Bi-LSTM	Forecasting
2022, [5]	Network throughput, Memory usage	Multivariate	DL	Bi-LSTM	Forecasting
2022, [12]	CPU utilization, Memory usage	Multivariate	DL	Deep neural network	Forecasting
2022, [22]	CPU utilization, Memory usage	Univariate/ Multivariate	-	-	Periodic Checking
2023, [7]	CPU utilization, Memory usage	Univariate/ Multivariate	-	-	Periodic Checking
Ours	Network throughput, Memory usage	Multivariate	DL	Bi-LSTM	Forecasting/Interim Checking

**Table 2:** Comparison of the time between prediction and interim inspection.

Phase	Time
Prediction	19.46 ms
Interim inspection	0.4528 ms

**Table 3:** Multi-bi-LSTM model configuration.

Hyperparameter	Values
Number of layers	2 layers (forward and backward)
Number of features	2
Input size	3
Number of hidden units per neuron	50
Loss function	MSE
Batch size	64
Epochs	100
Activation function	ReLU

**Table 4:** Estimation of autoscaling model with and without proposed post-processing algorithm.

Type	No Autoscaling	Autoscaling without Proposed Algorithm	Autoscaling with Proposed Algorithm
$\theta_{u}$ (%)	7.99	19.5	5.31
$\theta_{o}$ (%)	60.94	22.65	26.56
$\tau_{u}$ (%)	17.96	33.59	9.37
$\tau_{o}$ (%)	60.94	19.53	14.06
$\epsilon_{n}$ (%)	1.0	1.16	2.31

$\theta_{u}$ $\theta_{o}$ $\tau_{u}$ $\tau_{o}$ are metrics where smaller values are better; $\epsilon_{n}$ is a metric where larger values are better.

## Data Availability

Publicly available datasets were used in this study. The datasets can be found at GWA-T-12-Bitbrains (http://gwa.ewi.tudelft.nl/datasets/gwa-t-12-bitbrains, accessed on 19 August 2023).
